# The protected antimicrobial process in a University Teaching Hospital: a qualitative interview study exploring the knowledge, attitudes, and experiences of healthcare professionals

**DOI:** 10.1007/s11096-022-01381-z

**Published:** 2022-02-06

**Authors:** E. Burton, M. O’Driscoll, A. Fleming

**Affiliations:** 1 Pharmaceutical Care Research Group, School of Pharmacy, University College Cork, Cork, Ireland; 2 Pharmacy Department, Mercy University Hospital, Cork, Ireland

**Keywords:** Antimicrobial stewardship, Hospital setting, Protected antimicrobials, Qualitative

## Abstract

*Background* The protected or restricted supply of certain antimicrobials such as linezolid, caspofungin, aztreonam, in the acute hospital setting is an important element of Antimicrobial Stewardship (AMS) programmes to address the growing problem of antimicrobial resistance. This process involves submitting an application for use to be reviewed typically by a Consultant Microbiologist, Infectious Disease Consultant or Antimicrobial Pharmacist. *Aim* To investigate healthcare professionals’ knowledge, experiences, and attitudes towards the protected/restricted antimicrobials process in order to identify possible methods of optimisation and improvement. *Method* Semi-structured interviews with stakeholders involved in the protected/restricted antimicrobial prescribing, dispensing and administration process were conducted in September–October 2019 in a 350-bed voluntary, general, acute hospital in Ireland. Interviews were analysed by the Framework method and mapped to the Theoretical Domains Framework (TDF). *Results* Interviews were conducted with 8 Doctors, 4 Pharmacists and 3 Nurses. TDF domains identified included: ‘Knowledge’; ‘Social/professional role and identity’; ‘Social influences’; ‘Memory, attention and decision processes’; ‘Beliefs about consequences’; ‘Environmental contexts and resources’. The relationship between prescribers and the AMS Team was reported as a facilitator of the process, whereas the inconsistency of the filing and versions of forms on the wards were seen as challenges. *Conclusion* The results of this study have shown that the existing protected/restricted antimicrobial process is a multi-disciplinary effort with barriers that require attention in order to make future improvements. Standardization of the form across all wards, an electronic version of the form, and structured education around AMS were suggested to optimize the process.

## Impacts on practice

- Healthcare professionals practicing in the study site are aware of the protected/restricted antimicrobial process, the principals of antimicrobial stewardship that underpin it, and its benefits to patient care.
- In order to improve the protected/restricted antimicrobials process, standardization of the availability of the form in an electronic format is recommended to be introduced in the study hospital.
- Continued education, audit and feedback of the protected/restricted antimicrobial process with hospital healthcare professionals in the study site is essential to reinforce the impact of this Antimicrobial Stewardship (AMS) strategy.

## Introduction

Antimicrobial restriction or protection is an important and potential high impact Antimicrobial Stewardship (AMS) intervention in the hospital setting [1]. All Irish hospitals have been advised to introduce a policy of antimicrobial protection/restriction [1]. The antimicrobials which are restricted vary between hospitals, but typically include meropenem, linezolid, caspofungin and amphotericin B and several others. Formulary restriction/protection curtails the overuse of broad spectrum, costly and new antimicrobials [1, 2]. It establishes the requirement for local approval from an Infectious Disease physician, microbiologist, or other member of the AMS Team to sanction those antimicrobials [2]. The latter strategy may involve approval numbers or codes displayed in patient records, or charts, which illustrate approval being granted [2]. Ideally, a treatment plan is also included in the patient’s notes, outlining parameters such as duration of therapy [2]. Notably, these mechanisms are in addition to standard procedures, including the performance of culture tests to confirm causative pathogens and determine antimicrobial sensitivity profiles [2].

A systematic review of hospital AMS interventions found antimicrobial protection to be the most effective method to reduce the consumption of antimicrobials [3]. Rates of success for antimicrobial protection were found to be 66–87% [3]. Compared to persuasive practices (clinical intervention and feedback), restrictive practices (antimicrobial protection/restriction) had at least a three-fold greater effect in achieving AMS goals [3]. The benefits of antimicrobial restriction/protection include reduced antimicrobial consumption, reduced antimicrobial resistance, and reduced expenditure [4]. One study, involving 22 hospitals found that in those hospitals that restricted carbapenems, usage was consistently lower (approximately 20 days of therapy (DOT)/1000 patient days (PD)), depending on the year, than the hospitals that did not restrict carbapenems [5]. A recent meta-analysis found significant reductions in antimicrobial resistance after the introduction of antimicrobial protection, but only with non-fermenters (e.g., *Pseudomonas* species) [6]. Another study found that the incidence of all non-pseudomonal multidrug resistant (MDR) Gram-negative bacilli decreased significantly after carbapenem restriction, with that of extended spectrum beta lactamases (ESBL)-producing *Enterobacteriaceae* reducing from 10.87/1000 PD (number of MDR isolates per 1000 patient days) to 2.98/1000PD (*p* < 0.001) [7]. An antimicrobial cost reduction from €15,681 ± 1790 per month (total €78,409 in period 1) to €713 ± 256 per month (total €4.989 in period 2), following the introduction of antimicrobial protection/restriction, was achieved in another study [8].

There is a large body of published research investigating the impact of AMS interventions, however there is a lack of qualitative research investigating the antimicrobial restriction/protection process in the hospital setting. Existing qualitative studies focus on AMS interventions generally, with one study only considering the views of Infectious Disease Consultants, not other healthcare professionals [9]. The importance of qualitative enquiry to investigate current AMS behaviours and practices in the clinical setting has been widely recommended. Expansion of qualitative research has been recommended in order to enhance the understanding of factors influencing antimicrobial prescribing decisions and AMS practices [10–12]. In order to optimise the implementation of antimicrobial protection moving forward, it is necessary to explore the views and experiences of hospital healthcare professionals.

### Aim

This qualitative study aimed to investigate healthcare professionals’ knowledge, experiences, and attitudes towards the protected/restricted antimicrobials application process in an Irish hospital, and to identify possible methods of optimising and improving the process.

### Ethics approval

Ethical approval was granted by the Social Research Ethics Committee at University College Cork (on 23/07/2019). (Log 2019-009). Participants provided written informed consent.

## Method

### Study setting

The study hospital is a 350-bed voluntary general acute hospital, serving both public and private patients providing in-patient medical and surgical, day patient, outpatient, and emergency services. In the hospital, the process of antimicrobial protection is led by the AMS Team comprised of a Consultant Microbiologist, Infectious Disease Consultant and Antimicrobial Pharmacist. A hard copy of the restricted antimicrobial application form must be filled out by the prescribing doctor and the order approved by a member of the AMS Team, in order to use restricted antimicrobials.

Each ward has a Clinical Nurse Manager who is in charge of the ward, and a team of staff nurses. A Consultant is in charge of a team of junior doctors within their speciality (Registrar, Senior House Officer, Intern). Each ward is also assigned a Clinical Pharmacist.

### Study design

Semi-structured interviews with key stakeholders in the antimicrobial protection process were conducted from 13 September to 10 October 2019 by the primary researcher (EB). This approach facilitated in-depth exploration of participants’ views and experiences. [13, 14].

### Topic guide and interviewing

An interview topic guide (Table 1) was developed based on the study objectives, existing literature, and experience of the research team. Two pilot interviews, with a nurse and a pharmacist were conducted; these were included in the final analysis. The topic guide was then revised to include more probing questions and further exploration of participants’ responses.

**Table 1:** Summary of the interview topic guide

Area	Issues discussed
Demographic information	Profession, grade, gender
Knowledge of the process of antimicrobial protection	Experience with the process Awareness of the antimicrobials which are protected
Education and Training	Formal/informal training on the antimicrobial process Formal/informal training on AMS Antimicrobial guidelines
Interaction with the Antimicrobial Stewardship Team	Approval/denial of protected antimicrobial requests
Adherence to the protected antimicrobial process	Efficiency of the process Confidence executing the process Professional practices with protected antimicrobials (prescribing, dispensing, administering)

### Sampling

Purposive and convenience sampling were used to recruit participants in the hospital. The sampling strategy recruited doctors, pharmacists and nurses involved in implementing the restricted antimicrobial process in the hospital. This included members of the AMS Team. No other inclusion or exclusion criteria were applied. Participants were invited to participate in the study by face-to-face invitation on the wards. Written informed consent was obtained.

Twelve interviews were conducted (including the two pilot interviews) involving all required HCPs for representation. A further three interviews were conducted to ensure that data saturation had been reached and that no new themes emerged, as per the Francis method [15]. To support this, iterative data analysis begun in parallel with data collection. Interviews were audio-recorded and transcribed verbatim by the primary researcher. No repeat interviews were conducted. Field notes were handwritten after interviews.

### Analysis

Framework analysis was used to analyse the transcripts [16]. All transcripts were coded independently by one author (EB). QSR International’s NVivo 12 Qualitative Data Analysis Software was used to organise the coding. Initial, non-hierarchical codes were generated. These were reviewed in consultation with a second reviewer (MOD) with discussion of the final codes by all authors. Codes were then attributed to domains of the Theoretical Domains Framework (TDF) [16] (Table 2).

**Table 2:** Theoretical domains presented with explanatory definition and sample construct

Domain	Definition and example of a construct
Knowledge	An awareness of the existence of something, for example, procedural knowledge
Skill	An ability or proficiency acquired through practice, for example, competence
Social/professional role and identity	A coherent set of behaviours and displayed personal qualities of an individual in a social or work setting, for example, professional confidence
Beliefs about capabilities	Acceptance of the truth, reality or validity about an ability, talent, or facility that a person can put to constructive use, for example, self-confidence
Optimism	The confidence that things will happen for the best or that desired goals will be attained, for example, optimism, pessimism
Beliefs about consequences	Acceptance of the truth, reality, or validity about outcomes of a behaviour in each situation, for example, outcome expectancies
Reinforcement	Increasing the probability of a response by arranging a dependent relationship, or contingency, between the response and a given stimulus, for example, rewards
Intentions	A conscious decision to perform a behaviour or resolve to act in a certain way, for example, stability of intentions
Goals	Mental representations of outcomes or end states that an individual wants to achieve, for example, goal/target setting
Memory, attention and decision processes	The ability to retain information, focus selectively on aspects of the environment and choose between two or more alternatives, for example, decision-making
Environmental context and resources	Any circumstances of a person's situation or environment that discourages or encourages the development of skills and abilities, independence, social competence and adaptive behavior, for example, resources
Social influences	Those interpersonal processes that can cause individuals to change their thoughts, feelings or behaviours, for example, social pressure
Emotion	A complex reaction pattern, involving experiential, behavioural and physiological elements, by which the individual attempts to deal with a personally significant matter or event, for example, anxiety
Behavioural regulation	Anything aimed at managing or changing objectively observed or measured actions, for example, self-monitoring

Adapted from Cane et al. [16]

**Fig. 1:**
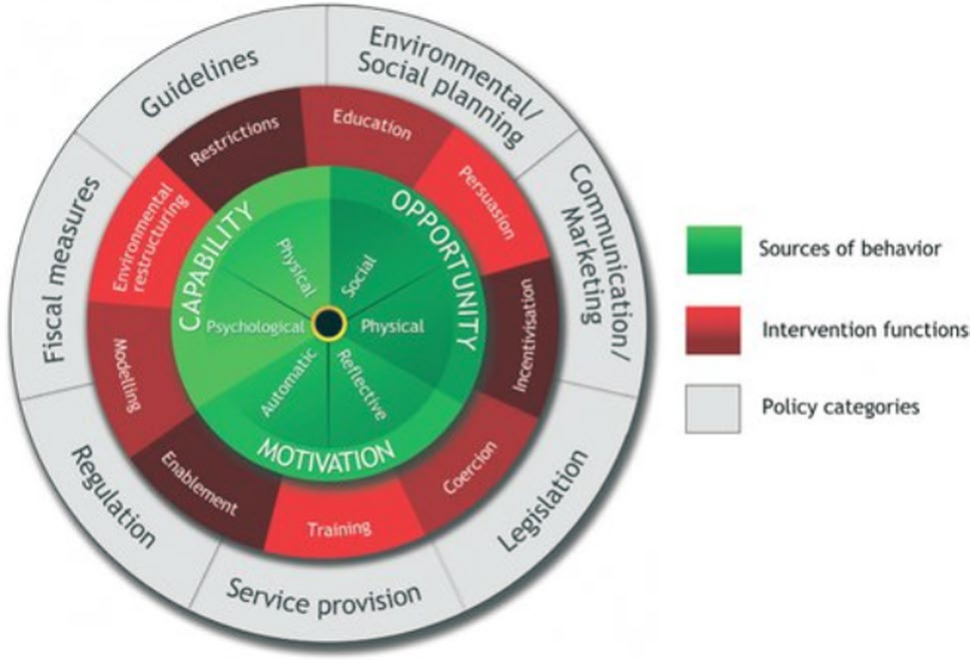
The Behaviour Change Wheel [17, 18]

The next stage involved identifying what behaviours needed to change and what methods could be recommended to achieve this. The TDF [16] domains were subsequently mapped onto the COM-B model and the behavioural change wheel (Fig. 1) [17, 18]. The COM-B model cites Capability, Opportunity and Motivation as three factors which can change behaviour [17–19]. The behaviour change wheel and COM-B, intervention functions, and behaviour change techniques (BCT v1) [20] were proposed to support the antimicrobial protection process going forward. The TDF, COM-B and BCTs have been previously used in qualitative research investigating AMS interventions [19, 21].

The study is reported in line with the checklist of the consolidated criteria for reporting qualitative research (COREQ) [22] and is presented in the supplementary information.

## Results

Fifteen interviews were conducted. Interview participants included: 8 Doctors, 4 Pharmacists and 3 Nurses. Participant detail is displayed in Table 3*.* The interviews ranged from 8 to 15 min. Key themes are presented by identifying the relevant TDF domains. Participant quotes are represented by their profession (doctor, pharmacist, nurse), and the corresponding number refers to their details in Table 3.

**Table 3:** Characteristics of participants interviewed in the study

Doctor	Grade	Specialty	Gender
1	Senior House Officer	Medical	F
2	Senior House Officer	Surgical	F
3	Consultant	Medical	M
4	Senior House Officer	Surgical	M
5	Intern	Surgical	F
6	Intern	Medical	F
7	Consultant	Medical	F
8	Intern	Surgical	F

Pharmacist	Grade		Gender
1	Senior Pharmacist or higher		M
2	Senior Pharmacist or higher		F
3	Basic Grade Pharmacist		F
4	Senior Pharmacist		F

Nurse	Grade		Gender
1	Clinical Nurse Manager (CNM1)	Medical/Surgical mix	F
2	Clinical Nurse Manager (CNM1)	Medical/Surgical mix	F
3	Clinical Nurse Manager (CNM2)	Medical/Surgical mix	F

### Theoretical domains framework

The analysis identified key domains of the TDF [16] that were found to be relevant, and they are described below. The other domains that were not identified (optimism, reinforcement, intentions, goals, and emotions) are not discussed as not enough references to the relevant constructs were made.

### Knowledge

Participants’ knowledge of the antimicrobial protection process was inconsistent. Most participants had a general understanding of the steps involved in the process and the reasons behind it. All participating pharmacists and nurses, and most of the medical team participants could identify examples of restricted antimicrobials, with meropenem, linezolid and caspofungin being mentioned most frequently. In all professions, apart from pharmacy, participants relayed that level of experience and knowledge influenced the individual’s interaction with the process. Senior nurses were involved with the process more frequently than staff nurses, and junior doctors filled out the forms more than Consultants. Some reported that a selection of their medical colleagues did not realize a form was required to order a restricted antimicrobial.

I’m not sure if the staff nurses on the ward would have as much experience in it [the process of antimicrobial protection], a lot of the time it’s being led by the senior nurses on the ward. (Nurse 3)

Most participants did not report engaging in any formal or informal AMS training, apart from their university education. Nursing staff stated that they receive “*on-the-job learning*” *(Nurse 2)* alone, in regard to this process.

Obviously as a pharmacist I would have training around antimicrobials, but in terms of the eh, I suppose the stewardship component of it, ehh nothing specialized. (Pharmacist 1)

### Social influences

The correct communication channels and procedures were identified by interviewees as being important components of the process. Doctors and nurses discussed contacting pharmacy or the AMS Team for advice. Pharmacists contacted microbiology before proceeding if any queries arose. Informal ward-based discussions between these three disciplines were also seen to positively influence adherence to the process. Participants all expressed how positive teamwork encouraged them to engage with the process. Recognition of the reliance on fellow professionals, especially those in more senior or specialised roles, in terms of tasks, knowledge or procedure, offered participants opportunities to clarify and question items surrounding antimicrobial prescribing.

It’s kind of a two-way street you know, there has to be a lot of working together. (Doctor 5)

Nursing and pharmacy appeared to drive the restricted antimicrobial process, and adherence to it, at ward and dispensary level. Nurses discussed presenting the forms to fill out when the medical team wished to prescribe a restricted antimicrobial. At dispensary level, pharmacy was seen by all disciplines as the “gatekeepers” of the restricted antimicrobials. At ward level, pharmacists encouraged the medical team to complete the form, and follow the correct procedure, or contacted the nurses to ask them to follow-up on the forms.

Pharmacy ensure that the forms are filled in completely and the supply is withheld until those forms are completed… (Pharmacist 1)

Those on the AMS Team spoke of the conflicts that can arise when medical opinions differ. Participants mentioned that at times the clinical opinion of a member of the AMS Team could contradict that of the Consultant ordering the restricted antimicrobial.

…people want to prescribe something and and I’ve been in opposition to it and that’s a major challenge at times (Doctor 3)

Other doctors spoke of factors which may lead to these discussions. The concept of the autonomy of the prescribing Consultant was discussed by junior doctors and members of the AMS Team. Acknowledgement that medical care and opinions can differ was evident.

At the end of the day like a consultant could do it whether the consultant microbiologist wants it or not you know what I mean?” (Doctor4)

However, the majority of junior doctors acknowledged that the decision of the AMS Team is always taken on board and usually accepted. Restricted antimicrobials were used more frequently by medical teams, rather than surgical teams. However, members of the AMS Team mentioned that they would encourage the surgical teams to be more aware of AMS in the context of surgical prophylaxis. Surgical junior doctors mentioned that they would not have filled out the restricted form if they were not prompted to do so by a phone call from the Consultant Microbiologist.

I think one area where we really, it would be nice if the message was accepted more it would be em antimicrobial prophylaxis in the surgical groups as I think that grips on for too long. (Doctor7)

### Memory attention and decision processes

All participants were aware that the process for applying for a restricted antimicrobial existed. However, participants relayed the stages of the process with varying degrees of familiarity. Members of the medical team in particular were unfamiliar with the process beyond the point of filling in the form. They were unsure of who collected the form, and its purpose when sent to pharmacy. Members of the nursing and pharmacy teams could relay all stages of the process.

Don’t know what happens once brought to pharmacy. But I think it’s to do with like them being able to order it in and stock and things like that. (Doctor 5)

The majority of participants believed that the process of antimicrobial restriction was superior in the study site than other hospitals. This was due to the interactive nature and strong ward presence of the AMS Team. However, others stated that they knew of other hospitals who were more stringent about withholding restricted antimicrobials. In most cases, pharmacy did not dispense the restricted antimicrobial unless the form was filled out. However, participants revealed that pharmacy was hesitant to leave a patient without a restricted antimicrobial, *“as the very nature of an antibiotic is you have to use it quickly” (Pharmacist 2).* Thus, at times a mutual understanding was reached. A form might be delivered the morning after receipt of the antimicrobial, due to clinical need, and availability of the AMS Team.

So, we can’t say we’re not giving it to you, we give them one vial of the antibiotic and we say to the nurses will ye go back and get the form for us before we can issue more. (Pharmacist 2)

### Beliefs about consequences

Overall, participants agreed that the restricted antimicrobial process led to positive patient outcomes. They reported that it improves appropriateness of treatment, reduces exposure to antimicrobial side effects, and reduces length of hospital stay, as well as preventing resistance developing to these antimicrobials. They acknowledged that AMS policies need to be adhered to in order to protect these antimicrobials.

You’re giving them the right antibiotic em for their particular indication so it would definitely contribute positively to patient care. (Pharmacist 4)

Most participants spoke of the forms and AMS programmes in the hospital as having a positive impact on prescribing practices. The medical team were aware that restricted antimicrobials are usually last line, and that escalating from other agents should only be done under certain circumstances. The form is the final step resulting from that decision, with review by microbiology.

You know there’s certain flags on the form that em, would would spur the doctor on to contact micro and discuss it and maybe not prescribe the restricted antibiotic so quickly in the first place. (Pharmacist 2)

A minority of participants, mostly those in more senior roles, referred to the process resulting in cost savings as restricted antimicrobials are typically more expensive. It was claimed that the process aids in *‘addressing cost related issues” (PH4).* The restrictive nature of the form acted like a *‘gatekeeper’ (Pharmacist 4).* The interviewees made it evident that the benefit to the patient had to be weighed up against the economic effects.

I suppose we particularly want to monitor the use of those, some of them from a cost point of view. Say particularly like the antifungals; like Ambisome is very expensive… (Doctor 7)

Conversely, a minority of professionals considered the possibility of negative patient outcomes. Some participants believed that the process prevents or slows down access to potentially critical antimicrobials. Other participants discussed the consequences for patients if their restricted antimicrobial regime was not reviewed by microbiology. They were concerned that patients may be prescribed the incorrect antibiotic or regime without specialist review.

…there had been some medications that the nurses couldn’t administer because the pharmacy hadn’t yet received the antimicrobial form ... (Doctor 1)

### Environmental contexts and resources

Although some participants believed the form itself to be a barrier to adherence to the process, difficulty locating the hard-copy forms was the primary logistical challenge reported by members of the medical teams. Some stated that there was an absence of forms on certain wards, with differing filing systems or form versions between wards.

Huge difficulty finding the forms; they’re different places on the wards. (Doctor 2).

There were actually two different forms. I wasn’t sure which one was right to use. (Doctor 5).

Restricted antimicrobials are stored in the pharmacy. However, participants from pharmacy and nursing mentioned that restricted antimicrobials stored on the wards, for a particular patient, at times were used for other patients, in breach of the process.

Patient A and B on the ward, and A was prescribed meropenem and it was appropriate, and we got the form, and everything was perfect, and we filed it away, but patient B then was prescribed it but because there was there was a supply of meropenem out on the ward already... The ward didn’t need to come to pharmacy for it so they were using that supply so there could be a day a day and a half of patient B getting meropenem and it could be totally inappropriate. (Pharmacist 2)

The alteration of the process out of hours was discussed by the majority of participants and was perceived as a challenge due to reduced access to the AMS Team. Nursing staff reported that the procurement of the antimicrobials out of hours is their responsibility, regardless of whether the form is filled in and approved by the AMS Team or not.

We’d normally either try to get it from another ward or the Assistant Director of Nursing would just have to open pharmacy. It’s something prescribed. We kind of have to do as much as we can to get it. Even if we don’t have the form. (Nurse 2)

Acquiring the form at a later date is seen to be the responsibility of pharmacy. Junior members of the medical team who needed to escalate a patient to a restricted antimicrobial out of hours, document the details in the chart and follow up with the AMS Team the following day. Members of the AMS Team stated that any restricted antimicrobial that is commenced out of hours will usually be followed up on within twelve hours. Some questioned the utility of twenty-four-hour cover for the AMS Team.

A call to me at three in the morning to authorize meropenem…? I mean really what I think we have to maybe work more towards is the education of our Non-Consultant Hospital Doctors. (Doctor 7)

### Social/professional role and identity

All participants were aware of their individual role in the process. Within professions, roles also differed depending on the grade of the healthcare professional, and their level of experience. All nurses interviewed stated that they assist in guiding the doctor through completing the form if they are unfamiliar with the process.

You know because I’d often be the one that would be liaising with pharmacy, so I am very I suppose I know what ones need the form now. (Nurse 2)

The nursing staff order the antimicrobials from pharmacy, administer them and review the duration of courses. Pharmacy is primarily involved in the process as the *“gate keepers “(Pharmacist 4)* at dispensary level, but also at ward level. Pharmacists check the form completeness and use their professional judgement to dispense the antimicrobial or not. The junior doctors liaise with the AMS Team on behalf of their team.

I feel my role is a link between you know passing on the patient information and consulting with the consultant. (Doctor 5)

### Application of BCT taxonomy and identification of potential intervention functions

The principles of the COM-B model, intervention functions and BCTs have been applied to the study findings to recommend strategies for improving and optimising the antimicrobial protection/restriction process in the study site (Table 4). The interventions listed in the table were suggested by or discussed with interviewees, thus improving future acceptability. Electronic systems may allow for more shared data in secondary care and more accurate comparisons with other hospitals, which could motivate healthcare professionals to reflect on and alter their prescribing patterns.

**Table 4:** Suggested intervention strategies identified by applying the TDF and BCT Taxonomy (V.1) to the study findings [20]

TDF Domain	COM-B	BCT taxonomy	BCT label	Strategy examples (with intervention function in italics)
Behavioral regulation Goals. Intentions Social/professional roles & identity	C—(Psych.) M—(Refl.)	Goals and Planning	Goal setting (outcome) Action planning Review outcome goals	*Enablement:* Streamline and standardize the process Implement an electronic version of the form
Knowledge Memory, attention, and decision-making processes Behavioral regulation Beliefs about capabilities Optimism	C—(Psych.) C—(Phys.) M—(Refl.)	Shaping knowledge, Natural consequences, Comparison of outcomes	Instructions on how to perform behavior Information about health consequences Credible source	*Education*: Structured AMS education sessions for all involved in the process Make available online recordings of the education sessions and up to date AMS information
Environmental context Memory, attention, and decision-making processes	O—(Phys.) C—(Psych.) C—(Phys.)	Antecedents, Associations	Restricting the physical environment Prompts/cues Adding objects to the environments	*Environmental restructure/enablement*: Dispose of older form versions on the wards Ensure all up-to-date forms are filed consistently in all wards
Knowledge. Memory, attention, and decision-making processes Behavioral Regulation Social influences	C-(Phys.) C-(Psych.) M-(Auto.)	Repetition and substitution	Behavioral practice/ rehearsal	*Training:* Structured training on the process
Goals Beliefs about Consequences and Capabilities Memory, attention and decision-making processes Behavioral Regulation. Social/professional roles and identity Social influences	M—(Refl.) C—(Psych.) O—(Soc.)	Feedback and Monitoring, Comparison of outcomes, Identity	Feedback on outcome of behavior. Discrepancy between current behavior and goal Incompatible beliefs Information about others’ approval. Social comparison	*Enablement*: Ensure frequent audit and feedback of the process *Persuasion*: Benchmark use of restricted antimicrobials of the study site against other hospitals
Reinforcement. Knowledge. Beliefs about Capabilities. Social/professional roles and identity	C—(Psych.) M—(Refl.)	Reward and threat, Scheduled consequences	Incentive (outcome) Reward approximation/ completion	*Incentivisation*: Positive reinforcement from AMS Team of audit results Potential financial savings if restricted antimicrobials consumption reduced

## Discussion

### Statement of key findings

This qualitative study has found that the process of antimicrobial restriction in one Irish university teaching hospital is a multidisciplinary effort between pharmacy, medicine, and nursing. The findings contribute valuable insights to the adherence and implementation of a key AMS strategy, which could be optimised to improve the protection of important antimicrobials. A key finding of this study was that knowledge of the process differed by the level of experience of the healthcare professional, and their healthcare discipline. Clinical Nurse Managers interacted with the process on a regular basis, signposting the medical teams towards the form and liaising with pharmacy about the procurement of the restricted antimicrobials. It was reported that staff nurses rarely engaged with the process. Within the medical teams, junior doctors were more familiar with filling in the forms than their consultant colleagues. However, this did differ depending on the speciality of the junior doctor. The study found that the daily implementation of the process varies, depending on whether or not there are forms on the ward, whether the request is made during working hours or out of hours, and in some cases, patients were reported as being administered these antimicrobials without full authorisation. Participants recommended that standardization of the process across all wards, an electronic version of the form, and formal education surrounding the process of antimicrobial protection and AMS were needed.

### Interpretation

#### Facilitators of and challenges to the antimicrobial protection process

All participants in this study cited the approachability of the AMS Team and resulting co-operation as facilitators to process adherence. This has been reported in previous studies, with an Australian survey study citing enablers to prescribing such as ‘an acknowledgement of the need for assistance in prescribing’ and reported readiness to consult local and prescribing guidelines [23]. All participants spoke of the positive relationship between clinical staff on the wards, and the AMS Team. Effective teamwork is globally recognised as a necessary tool in the construction of a patient-centred and effective healthcare system [24–26]. The majority of challenges related to the process were consistently reported as issues that were practical and solvable, including the location of the most recent version of the forms on the wards. However, it was also reported that on certain occasions, such as out of hours, a patient could be given a restricted antimicrobial agent approved for another patient due to factors such as unavailability of the AMS Team. This issue could be seen as more complex and would require a more intricate solution. The literature highlights that interventions tailored to address specifically identified barriers are more likely to change behavior and improve professional practice when it comes to AMS programmes [27].

#### Proposed improvements to the process

Different versions of the restricted antimicrobial forms were reported as being present on the wards. Old versions on the wards and inconsistent filing between wards was discussed frequently. Previous studies have found that implementing evidence-based interventions in a uniform manner within a hospital can be a huge challenge [28].

Participants felt that the process itself was necessary, but that it could be made more efficient, on a practical level. Standardization of clinical documentation has been emphasised recently in order to improve processes in healthcare settings [28–30] Standardising the form, removing old versions, or moving to an electronic version and application process would improve the process. Participants from medicine and nursing believed that an electronic version of the restricted antimicrobial form that would be sent directly to pharmacy would be more efficient and reduce the time taken to approve the antimicrobial and administer it to the patient. The literature confirms that electronic forms in healthcare ensure accurate data collection, easy manageability, consistent form rendering and interoperability [31]. The introduction of computerized restricted antimicrobial forms resulted in a reduction in antibiotic consumption [32, 33] in two studies and an increase in compliance with the process of antimicrobial protection, surveillance and restricted antimicrobial approvals obtained within 24 h in another study [34]. However, further literature reports that a common reason for the failure of electronic solutions in healthcare is that clinicians will not use the technology [35]. It has been recommended that co-design of health technologies should be carried out to facilitate usability and acceptance [36].

#### Education

All participants spoke about restriction reducing the emergence of resistance. This is a well-known reason for implementing AMS strategies such as restriction, and is highly supported by the literature [1, 5, 37]. Participants relayed that any AMS training they received was as part of their undergraduate degrees, “on-the-job” learning and through “grand rounds” in the study site. The medical team also discussed teaching sessions with members of the AMS Team. Most interview participants suggested the introduction of a structured AMS education programme in the hospital which would help to standardise knowledge around restricted antimicrobials, antimicrobial resistance and improve the restricted antimicrobials process. There is substantial evidence for the efficacy of these programme and their acceptability amongst clinicians [38]. Further evidence suggests that education programmes should be implemented in the context of an active hospital AMS Team who are responsible for overseeing restrictive and supportive AMS interventions [39].

#### Strengths and weaknesses

A limitation of this study was that participants were recruited from one hospital. Conducting a multi-site study would provide more broadly applicable and nationally representative results. However, the challenges faced by this study site are likely to be common across the Irish hospital setting as mentioned by some participants. Furthermore, interview length was relatively short, due to the clinical commitments of staff. Despite this, data saturation was reached as this was a focused topic of discussion and the interviews generated detailed accounts of the attitudes, knowledge and experiences of staff involved in the process of antimicrobial protection in the study site. The interviewer was known in a professional capacity by some participants; she presented herself as a researcher, and the open and insightful views of participants reassures the research team that this did not increase the risk of social desirability bias. The study had a limited sample size, however the inclusion of participants from different healthcare professional groups increases the representation of the findings. Similarly, the prevalence and consistency of findings, is outlined in the results.

#### Further research

This study has identified potential areas for future implementation and the importance of qualitative investigation of AMS initiatives. This has been highlighted in recent papers recommending that qualitative research efforts be strengthened in order to understand the behavioral determinants involved in AMS initiatives and strategies [12, 40]. Standardising and streamlining the process across wards through administrative, clinical and governance channels could be investigated. The possibility of electronic restricted antimicrobial forms could be explored. Additionally, more formal Antimicrobial Stewardship education programmes could be rolled out across secondary care institutions.

## Conclusion

The results of this study have shown that the existing restricted antimicrobial restriction process is a multidisciplinary effort. Process improvements to standardize the process across all wards, an electronic version of the form, and education to raise awareness were recommended to improve the implementation of this AMS strategy. Although this single site study has limitations, it offers valuable insights into the knowledge and attitudes of healthcare professionals in relation to restricted antimicrobials. It also highlights variable levels of adherence to the policy, and room for improvement in this regard. Findings may inform future initiatives in other locations for improved outcomes.
